# Dental morphology of the Lesser Bamboo Rat, *Cannomys badius* (Rodentia, Spalacidae)

**DOI:** 10.3897/zookeys.228.3826

**Published:** 2012-10-17

**Authors:** Raquel López-Antoñanzas

**Affiliations:** 1 Departamento de Paleobiología, Museo nacional de Ciencias naturales–CSIC, C/ José Gutiérrez Abascal 2, 28006 Madrid, Spain

**Keywords:** Rhizomyinae, Rhizomyini, *Cannomys*, dental wear pattern

## Abstract

*Cannomys* and *Rhizomys* are the sole living genera of the tribe Rhizomyini (Rhizomyinae, Spalacidae, Rodentia), known in the fossil record since the Late Miocene. The dental morphology of fossil Rhizomyini has been described in detail but until recently such descriptions were unavailable for extant species. A detailed account of the morphology and dental wear pattern of the cheek teeth of *Cannomys badius* is provided here based on the examination of museum specimens. Three stages of wear are recognized. *Cannomys* shares with *Rhizomys* the synapomorphy of having a mesolophid that is a long continuation of the protoconid on the first lower molar. There are significant differences between these taxa, such as the much smaller size of the cheek teeth and the trilophodont dental pattern of the M2, M3, and m2 in *Cannomys*.

## Introduction

The subfamily Rhizomyinae (Spalacidae, Rodentia) is known in the fossil record since the Oligocene. It is represented by three modern genera: the Asian bamboo rats *Rhizomys* (a trispecific genus) and *Cannomys* (monospecific) and the African mole rats *Tachyoryctes*. The latter genus is considered bispecific by some authors (Missone 1971, Rahm 1980, Bucher 1982, Corbet and Hill 1991, Beolchini and Corti 2004), but this may be an underestimation of true diversity (Allen 1939, Ellerman 1941, Musser and Carleton 1993, 2005). The Asian bamboo rats belong to the tribe Rhizomyini, whereas *Tachyoryctes* is the only extant representative of the Tachyoryctini (Flynn 2009; López-Antoñanzas et al. in press). The lesser bamboo rat *Cannomys badius* ranges from eastern Nepal, through northeast India, Bhutan, southeastern Bangladesh, Myanmar, south China, northwest Vietnam, Thailand and Cambodia (Musser and Carleton 2005). It is known to live in a wide variety of habitats, from bamboo forest to cultivated land and other modified areas (IUCN 2010).

*Cannomys badius* was originally named as *Rhizomys badius* by Hodgson (1841; p. 60) on the basis of a male specimen obtained “some miles north of the great valley” of Nepal. The type specimen is BMNH 1843.1.12.61 (skin and skull) (P. Jenkins, pers. comm. 2012). Thomas (1915a) created the new genus *Cannomys* for which he took *Rhizomys badius* as type, but in which he also included two other species: *Cannomys castaneus* (Blyth 1843) and *Cannomys minor* (Gray 1842). Thomas (1915b) later described another species of *Cannomys*, *Cannomys pater*, and split *Cannomys castaneus* into two subspecies (*Cannomys castaneus castaneus* and *Cannomys castaneus plumbescens*). Later, Gyldenstolpe (1916) described a new subspecies of *Cannomys minor* (*Cannomys minor lönnbergi*). All these taxa are currently regarded as synonyms of *Cannomys badius* (Musser and Carleton 2005).

The dental morphology of all living Rhizomyinae other than *Cannomys badius*, has been described in detail recently (*Tachyoryctes*; López-Antoñanzas 2012) or are in the process thereof (*Rhizomys*). The aim of the present work is to provide an in-depth description of the dental morphology of *Cannomys*.

## Material and methods

All the specimens of *Cannomys badius* housed in the Laboratoire de Zoologie-Mammifères et Oiseaux of the Muséum national d’Histoire naturelle, Paris, France (MNHN) and in the Institut für Systematische Zoologie-Zoologische Sammlung of the Museum für Naturkunde, Berlin, Germany (ZMB) have been examined. MNHN individuals come from Thailand and Nepal, whereas those at ZMB are from Myanmar.

**Figure 1.**
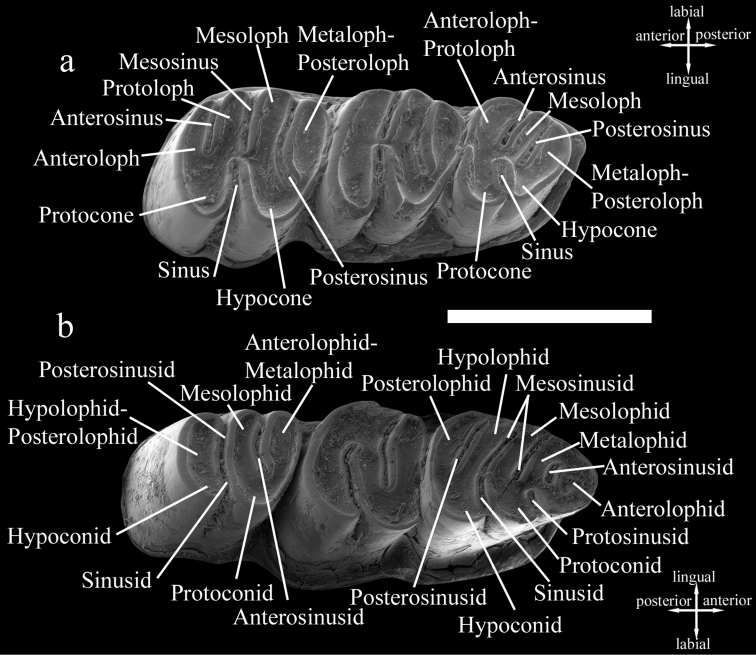
Dental terminology used in this paper. *Cannomys badius*. **a** Left upper cheek teeth **b** Right lower cheek teeth. Scale bar equals 5 mm.

First, second, and third lower molars are designated as m1, m2, and m3, respectively, and first, second, and third upper molars as M1, M2, and M3, respectively. The terminology used in the tooth descriptions follows the rodent dental terminology of Flynn (1982) with some adjustments (see Fig. 1).

### Dental morphology and wear pattern

Incisors of *Cannomys badius* are strongly proodont, flattened anteriorly, lack major ornamentation and have the enamel pigmented orange.

**Figure 2.**
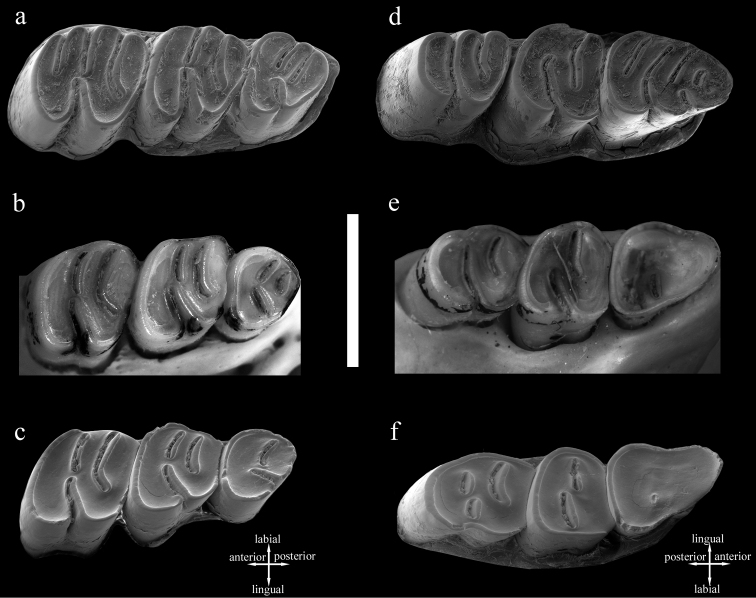
Dental wear pattern in *Cannomys badius*. **a–c** Upper molars: **a** Stage of wear 1, juvenile individual, left maxilla with M1-M3 in occlusal view (MNHN C.G. 1860-382) **b** Stage of wear 2, left maxilla with M1-M3 (ZMB 44769) **c** Stage of wear 3, left maxilla with M1-M3 in occlusal view (MNHN C.G. 2000-761). **d–f** Lower molars: **d** Stage of wear 1, juvenile individual, right hemimandible with m1-m3 in occlusal view (MNHN C.G. 1860-382), **e** Stage of wear 2, right hemimandible with m1-m3 in occlusal (ZMB 44768) **f** Stage of wear 3, left hemimandible with m1-m3 in occlusal (reversed) (MNHN C.G. 2000-761). Scale bar equals 5 mm.

The upper molars of *Cannomys badius* show unilateral hypsodonty, with crowns higher lingually than labially. m1 has four roots, the anterolabial one being the most developed. Its occlusal outline is square. In early wear (e.g., MNHN C.G. 2000-271; Fig. 2a), it has four transverse lophs (anteroloph, protoloph, mesoloph, and metaloph-posteroloph) and all labial and lingual sinuses are open. In later wear (e.g., ZMB 44769 and MNHN C.G. 2000-761; Fig. 2b–c), the number of lophs is reduced to three as the anteroloph and protoloph combine and join at the margin of the tooth, isolating two transversely elongated enamel lakes, whereas the lingual sinus remains open. The latter, narrow and short, is directed toward the middle enamel lake. The connection between the anterior and posterior parts of the tooth persists through wear.

The occlusal outline of m2is square, with its posterior side more reduced than the anterior one. This tooth is much shorter than m1. In early wear (e.g., MNHN C.G. 1860-382; Fig. 2a), it has three transverse lophs (anteroloph-protoloph, mesoloph, and metaloph-posteroloph). The sinus is directed toward the anterosinus. All reentrants remain open. After moderate wear (e.g., ZMB 44769, Fig. 2b), the sinus becomes narrower and the anterosinus and posterosinus are closed-off, isolating two enamel lakes. Late in wear (e.g., MNHN C.G. 2000-761; Fig. 2c), the morphology of M2 is quite similar to that in the previous wear stage. However, the posterior part of the tooth becomes more reduced and, even though the lingual sinus remains open, it turns out to be more anterolabially directed due to the labial displacement of the hypocone. The connection between the anterior and posterior parts of the tooth persists through wear.

*Cannomys* has a reduced m3. In early wear (e.g., MNHN C.G. 1860-382; Fig. 2a), it is morphologically similar to m2 but with its posterior part smaller due to the more labial position of the hypocone. This tooth is trilophodont, with anteroloph-protoloph, mesoloph, and metaloph-posteroloph. After moderate wear (e.g., ZMB 44769; Fig. 2b), the anteroloph-protoloph is nearly connected to the mesoloph and the metaloph-posteroloph joins the mesoloph, isolating a labial circular enamel lake. The hypocone is much more labially displaced and the sinus much more anterolabially oriented. Therefore, the posterior part of the tooth becomes much reduced. In late wear (e.g., MNHN C.G. 2000-761; Fig. 2c), the first and second lophs combine and the anterior enamel lake disappears. At this stage of wear only a labial enamel lake persists and the hypocone is located on the posterior margin of the tooth.

The lower molars are lower crowned than the upper molars. As for the lower jaw bone, the mandibular foramen is located well caudal to the posterior margin of m3 (a little dorsal to m3, at the level of the tip of the coronoid process), whereas the mental foramen is situated rostrally to the anterior border of m1 (approximately on the midline of the dentary).

The occlusal outline of m1 is triangular, with its anterior part much narrower than its posterior. In early wear (e.g., MNHN C.G. 1860-382; Fig. 2d), it shows a pentalophodont dental pattern with anterolophid, metalophid, mesolophid, hypolophid, and posterolophid. The metalophid joins lingually the anterolophid and labially the protoconid, isolating a small and oval anterior enamel lake. The mesolophid is a long continuation of the protoconid. The anterior part of the tooth is isolated from the rest of the crown by a long sinusid. The latter results from the junction of the sinusid (or labial reentrant) with the mesosinusid (or middle labial reentrant). All lophids join the lingual margin of the tooth, isolating four transversely elongated enamel lakes. The m1 shows two open labial sinusids (protosinusid and sinusid). After moderate wear (e.g., ZMB 44768; Fig. 2e), all reentrants are closed-off, the protosinusid disappears, and the anterior part of the tooth is deprived of any enamel lake. However, two enamel lakes persist posteriorly. In late wear (e.g., MNHN C.G. 2000-761; Fig. 2f), the occlusal surface is completely flat and generally devoid of enamel lakes although a tiny and circular posterolabially located enamel lake may persist. The enamel has disappeared from the anterior border of the tooth, but it persists posteriorly.

The m2 has four roots, the posterior ones being the most developed. Its occlusal outline is square and it is anteroposteriorly compressed. In early wear (e.g., MNHN C.G. 1860-382, Fig. 2d); this tooth has three lophs (anterolophid-metalophid, mesolophid, and hypolophid-posterolophid). The mesolophid is short and joins labially the anterior lophid (through the protoconid) and lingually the posterior one. The sole lingual reentrant is closed-off, isolating a labial enamel lake, which is elongated. The narrow and posterolingually directed sinusid is open. After moderate wear (e.g., ZMB 44768, Fig. 2e); the morphology of the tooth is similar to that of the preceding stage. In late wear (e.g., MNHN C.G. 2000-761; Fig. 2f), the sinusid is closed off and the tooth shows both a labial and a lingual enamel lake. The anterior side of the tooth loses the enamel.

In early wear (e.g., MNHN C.G. 1860-382; Fig. 2d), m3 has three lophids (anterolophid-metalophid, mesolophid, and hypolophid-posterolophid). The first lophid joins the second one through the protoconid. The posterior lophid is isolated from the rest of the crown by a long reentrant. At this stage of wear, all reentrants are open. After moderate wear (e.g., ZMB 44768, Fig. 2e), the lingual reentrants are closed-off. The tooth has an elongate anterior enamel lake and a long sinusid. In late wear (e.g., MNHN C.G. 2000-761; Fig. 2f), the lingual reentrant is closed-off, isolating two enamel lakes, and the anterior enamel lake persists.

## Conclusion

The examination of the cheek teeth in various specimens of *Cannomys badius* has allowed determining the changes undergone by the dental pattern during wear. Three fundamental stages of wear have been recognized.

As postulated by Flynn (1990), modern and extinct rhizomyines are united by dental features that include lophodonty, high crowned teeth, a strong wear gradient decreasing from first to last molar, junction between the metalophid and the anterolophid or the protoconid on the lower molars, and isolation of enamel lakes. In addition, *Cannomys* shares with *Rhizomys*, the other extant representative of the tribe Rhizomyini, the synapomorphy of having on the m1 a mesolophid that is a long continuation of the protoconid. However, *Cannomys badius* is much smaller than all the extant species of *Rhizomys* and exhibits the particularity of a trilophodont dental pattern on m2, M2, and M3, whereas it is tetralophodont in *Rhizomys*.
